# Xeroderma pigmentosum

**DOI:** 10.1186/1750-1172-6-70

**Published:** 2011-11-01

**Authors:** Alan R Lehmann, David McGibbon, Miria Stefanini

**Affiliations:** 1 Genome Damage and Stability Centre, University of Sussex, Falmer, Brighton BN1 9RQ, UK; 2 Department of Photodermatology, St Thomas' Hospital, Westminster Bridge Road, London SE1 7EH, UK; 3 Istituto di Genetica Molecolare, Consiglio Nazionale delle Ricerche, Via Abbiategrasso, 207, 27100 Pavia, Italy

## Abstract

Xeroderma pigmentosum (XP) is defined by extreme sensitivity to sunlight, resulting in sunburn, pigment changes in the skin and a greatly elevated incidence of skin cancers. It is a rare autosomal recessive disorder and has been found in all continents and racial groups. Estimated incidences vary from 1 in 20, 000 in Japan to 1 in 250, 000 in the USA, and approximately 2.3 per million live births in Western Europe.

The first features are either extreme sensitivity to sunlight, triggering severe sunburn, or, in patients who do not show this sun-sensitivity, abnormal lentiginosis (freckle-like pigmentation due to increased numbers of melanocytes) on sun-exposed areas. This is followed by areas of increased or decreased pigmentation, skin aging and multiple skin cancers, if the individuals are not protected from sunlight. A minority of patients show progressive neurological abnormalities. There are eight XP complementation groups, corresponding to eight genes, which, if defective, can result in XP. The products of these genes are involved in the repair of ultraviolet (UV)-induced damage in DNA. Seven of the gene products (XPA through G) are required to remove UV damage from the DNA. The eighth (XPV or DNA polymerase η) is required to replicate DNA containing unrepaired damage. There is wide variability in clinical features both between and within XP groups. Diagnosis is made clinically by the presence, from birth, of an acute and prolonged sunburn response at all exposed sites, unusually early lentiginosis in sun-exposed areas or onset of skin cancers at a young age. The clinical diagnosis is confirmed by cellular tests for defective DNA repair. These features distinguish XP from other photodermatoses such as solar urticaria and polymorphic light eruption, Cockayne Syndrome (no pigmentation changes, different repair defect) and other lentiginoses such as Peutz-Jeghers syndrome, Leopard syndrome and Carney complex (pigmentation not sun-associated), which are inherited in an autosomal dominant fashion. Antenatal diagnosis can be performed by measuring DNA repair or by mutation analysis in CVS cells or in amniocytes. Although there is no cure for XP, the skin effects can be minimised by rigorous protection from sunlight and early removal of pre-cancerous lesions. In the absence of neurological problems and with lifetime protection against sunlight, the prognosis is good. In patients with neurological problems, these are progressive, leading to disabilities and a shortened lifespan.

## Review

### Disease name

Xeroderma pigmentosum (XP); Orpha 910

### Definition

Xeroderma pigmentosum (literally dry pigmented skin), is defined by extreme sensitivity to sunlight, resulting in sunburn, pigment changes in the skin and a greatly elevated incidence of skin cancers. About 60% of affected individuals show an exaggerated and prolonged sunburn response. In a minority of cases there are neurological abnormalities of varying severity. Historically, the disorder was classified originally as "classical XP" (skin abnormalities only) and the De-Sanctis-Cacchione syndrome with skin abnormalities and extreme neurological degeneration. The latter term is currently rarely used as it is evident that there is a wide range of neurological abnormalities of varying severity and varying age of onset. Thus, the complete De Sanctis-Cacchione syndrome is present in only very few cases, but several patients have one or more neurological features.

### Epidemiology

XP has been found in all continents and across all racial groups. Consistent with autosomal recessive inheritance, males and females are similarly affected. Estimates made in the 1970's suggested an incidence in the USA of 1 in 250, 000 [1] and in Japan of 1 in 20, 000 [2]. A more recent survey in Western Europe suggests approximately 2.3 per million live births [3]. Anecdotally, the incidence in North Africa and the Middle East, where there is a high level of consanguinity, is substantially higher.

### Clinical Description

There are many reviews of the clinical features of XP [1,4-6]. In about 60% of cases, the first signs are extreme sensitivity to sunlight [6], which takes many days or weeks to resolve. In these individuals, this sunburn reaction can happen in the first weeks of life and is often blamed on neglect or labelled wrongly as cellulitis or impetigo. The other 40% of cases do not show any sunburn reaction. In these cases, the first manifestation, often by two years of age, is an unusually increased number of lentigines (freckle-like pigmentation) in sun-exposed areas. They are present on the nose, zygoma and forehead and then appear on the sides of the neck, sparing the area under the chin. Photophobia is often present. In the absence of sun protection, the skin ages, becoming dry, rough and atrophic. Lentigines increase in number and darken and are difficult to distinguish clinically from the many, flat, pigmented seborrhoeic warts, which also proliferate and become warty. Small, hypopigmented macules are commonly seen amongst the lentigines and may even be the first presentation. Telangiectasia can be a late feature. Stucco keratoses may be present and are readily distinguishable from solar keratoses. As all the skin changes are the result of exposure to UV radiation, the severity of these changes is absolutely dependent on the amount of sun-exposure, the Fitzpatrick skin type and the degree of protection of the skin from sunlight. The effects vary a great deal between individuals. In the absence of rigorous protection from the sun, areas of hyper- and hypo-pigmentation will result, followed by accelerated photo-ageing, warty lesions, in-situ melanocyte and keratinocyte malignancy, and eventually multiple basal cell carcinomas and invasive squamous cell carcinomas and melanomas. It has been estimated that XP patients have a 10, 000-fold increased risk of non-melanoma skin cancer and a 2, 000-fold increased risk of melanoma under the age of 20 [6]. The early age of onset and the frequency of skin cancers in an otherwise normal individual should trigger further assessment for XP. In addition to the very large increase in skin cancer there is an approximately 50-fold increase in internal neoplasms, especially of the central nervous system.

Ocular abnormalities are almost as common as the cutaneous abnormalities, but they are strikingly limited to the anterior, UV-exposed structures of the eye (lids, cornea, and conjunctiva). Photophobia is often present and may be associated with prominent conjunctival injection. Continued sunlight exposure may result in severe keratitis, leading to corneal opacification and vascularization, and in neoplasms (epithelioma, squamous cell carcinoma, and melanoma) [5,7].

In XP patients there is also a greatly increased frequency of cancer of the oral cavity, particularly squamous cell carcinoma of the tip of the tongue, a presumed sun-exposed area [5].

20-30% of patients have neurological problems and intellectual deficiency [1,6,8]. The time of onset can vary from the age of two to middle age. The neurological abnormalities are the result of progressive neuronal degeneration resulting in sensorineural deafness, ataxia, areflexia, microcephaly and intellectual deficiency as well as impaired eyesight.

The clinical features are dependent on exposure to sunlight, the complementation group, the precise nature of the mutation as well as unknown factors. Consequently there is a huge variation in clinical features. Sunny climates, outdoor living, fair skin, smoking, poor availability of diagnostic facilities, delayed diagnosis and poor protection from sunlight will exacerbate the cutaneous features, resulting in multiple pigmentation changes, multiple skin cancers and early death. Conversely, less sunny climates, indoor living, pigmented skin, early diagnosis and good solar protection, can result in relatively mild skin features. However even allowing for these factors, there is much variability, especially in cases that do not show the acute sunburn reaction. The abnormal freckling may become apparent by the age of two, but may occur much later.

It might be expected that the patients with the most severe repair defects would show the most extreme sunburn reactions and the highest incidence of skin cancer. Paradoxically, however, those patients with acute sunburn reactions develop fewer skin cancers than those who do not. This is probably the result of earlier diagnosis of the former group and a disinclination of this group to go out in sunlight without protection.

The XP variant (XP-V) sub-type refers to patients with a somewhat different molecular defect (see below). These patients may display relatively mild features, but some have severe skin problems. Individuals with XP-V do not have neurological problems and, like those with XP-C, their sunburn pattern conforms to their skin type.

There are a few rare cases with combined features of XP and Cockayne Syndrome. These individuals have the severe neurological and developmental problems characteristic of Cockayne Syndrome together with the solar-induced pigmentation changes of XP.

### Aetiology

XP is an autosomal recessive disorder with 100% penetrance and can result from mutations in any one of eight genes. The products of seven of these genes (XP-A through G) are involved in the repair of ultraviolet-induced photoproducts in DNA by the process of nucleotide excision repair (NER) [5]. The XPC and XPE proteins are needed to recognise the photoproducts in DNA. XPB and XPD are part of a protein complex TFIIH, which opens up the structure of the DNA around the site of the photoproduct. XPA protein verifies that proteins are in the correct position and then the nucleases XPG and XPF cut the DNA on either side of the damage, so that the damaged section can be removed and replaced with intact DNA.

There are two branches of NER, designated transcription-coupled repair, which rapidly repairs areas of DNA that are "active" and being transcribed into RNA, and global genome repair, which repairs damage in the rest of the genome more slowly. XPC and XPE proteins are only required for the latter branch, whereas all the other XP proteins are required for both branches. Probably as a consequence of this, patients defective in the *XPC *or *XPE *genes do not, in general, have the extreme sunburn reactions or neurological abnormalities described above.

Defects in the eighth XP gene do not affect NER. Instead these so-called XP variants (XP-V) have problems replicating DNA containing ultraviolet-induced damage [9]. DNA replication is carried out by DNA polymerases. The DNA polymerases that normally replicate DNA cannot deal with damage in the DNA template and specialised polymerases have to be employed to get past the damage (translesion synthesis). For UV damage, the cell uses DNA polymerase η, encoded by the gene *POLH *and it is this gene that is mutated in XP-V patients [10]. Like XP-C and XP-E patients, XP-V patients rarely have extreme sunburn reactions or neurological problems.

The genes, chromosomal locations and the functions of the protein products are listed in Table 1.

**Table 1:** The XP genes

Gene	No of exons	**Chromosomal location**^**a**^	Protein size (aa)	Protein function	Defective pathway
***XPA***	6	**9q22.33**	**273**	**Damage verification**	**NER**

***XPB/ERCC3***	15	**2q14.3**	**782**	**Helicase**	**NER**

***XPC***	16	**3p25.1**	**940**	**Damage recognition**	**NER (GGR)**

***XPD/ERCC2***	23	**19q13.32**	**760**	**Helicase**	**NER**

***XPE/DDB2***	10	**11p11.2**	**427**	**Damage recognition**	**NER (GGR)**

***XPF/ERCC4***	11	**16p13.12**	**916**	**Nuclease**	**NER**

***XPG/ERCC5***	15	**13q33.1**	**1186**	**Nuclease**	**NER**

***XPV/POLH***	11	**6p21.1**	**713**	**Polymerase**	**TLS**

Abbreviations. NER: Nucleotide excision repair; GGR: global genome repair sub-pathway; TLS: translesion synthesis.

^a ^From [23].

The molecular defects in XP cells result in a greatly elevated induction of mutations in sun-exposed skin of affected individuals. This increased mutation frequency probably accounts for the pigmentation changes and the skin cancers. Indeed examination of mutations in the p53 gene in tumours from XP patients reveal p53 mutations characteristic of UV exposure in the majority of tumours [11]. The molecular defect also results in increased UV-induced lethality, which varies substantially between individuals. The level of cell killing is less in individuals mutated in the *XPC *and *XPE *genes and with some hypomorphic mutations in other XP genes, because of the residual functional DNA repair. These individuals also do not show the sunburn reaction found in other groups. This has led to the suggestion that the extreme sunburn reaction is likely to be a consequence of cell death.

The causes of the neurological abnormalities are poorly understood. They are clearly not connected with exposure to UV light. Current theories suggest that oxidative DNA damage is generated during normal metabolism in the central nervous system, and that some types of this damage must be repaired by NER [12]. In the absence of functional repair, the lesions persist and result in neuronal death.

### Diagnosis

**Figure 1:**
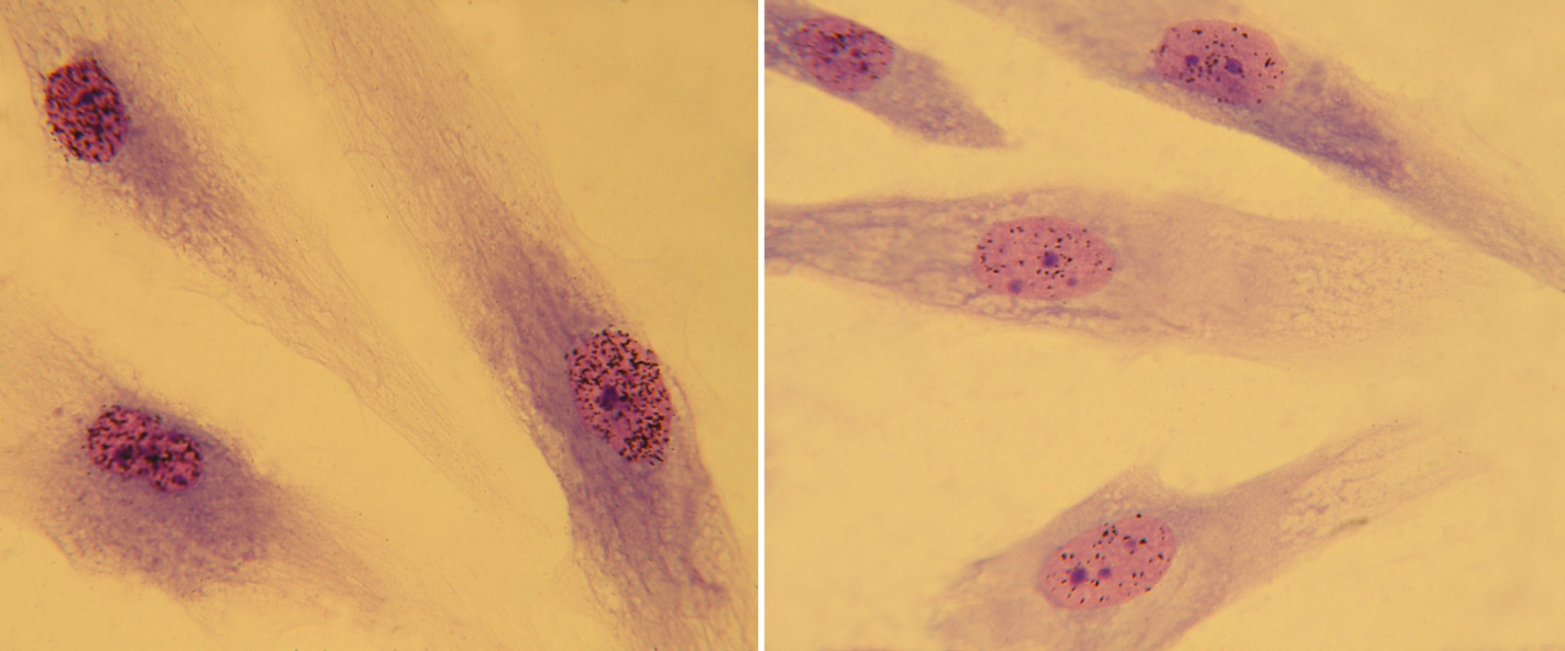
**UDS assay**. UV-induced DNA repair synthesis (unscheduled DNA synthesis, UDS) on autoradiographic preparations of primary dermal fibroblasts irradiated with UV light and incubated with ^3^H-thymidine, a DNA radioactive precursor. The labelling pattern in G1 and G2 nuclei reflects the amount of precursor incorporated during repair synthesis. Therefore, the number of grains is a direct and quantitative measurement of the ability of the cell to perform excision repair. Compared to normal cells (left panel), cells from a XP patient (right panel) show fewer grains and, thus, a reduced ability to perform UDS (courtesy of Tiziana Nardo, IGM CNR Pavia).

In most cases, the initial clinical diagnosis can be made on the basis of either the extreme sensitivity to UV in those individuals who show this feature, or in the appearance of lentiginosis on the face at an unusually early age. The diagnosis can be confirmed definitively by employing robust cellular tests for defective DNA repair that are available in several countries. The most commonly used test is the measurement of unscheduled DNA synthesis in cultured skin fibroblasts. After DNA damage has been removed, a patch of newly-synthesised DNA replaces the damaged section. Synthesis of this new DNA has some different features from synthesis of DNA during normal replication and the former is therefore referred to as unscheduled DNA synthesis or UDS. Skin fibroblast cultures are established from a 3-4 mm punch biopsy taken from an unexposed area of the skin, such as the upper, inner arm or the buttocks. Fibroblasts are UV-irradiated in a Petri dish, and UDS can be measured as incorporation of nucleotides into DNA of the irradiated cells either by autoradiography [13] (Figure 1) or liquid scintillation counting [14], or more recently using a fluorescence assay [15]. A reduced level of UDS confirms the diagnosis of XP.

Individuals with XP-V do not show this defect in UDS, as NER is unaffected in these patients. Furthermore XP-V cells are not hypersensitive to killing by UV light. However, it has been found empirically, that caffeine specifically sensitises XP-V cells to killing by UV [16]. To diagnose XP-V cells, cultures are exposed to UV, incubated in caffeine for a few days and their viability compared to that of normal cells. Specific sensitivity to UV in the presence of caffeine together with normal UDS confirms the diagnosis of XP-V [17].

**Figure 2:**
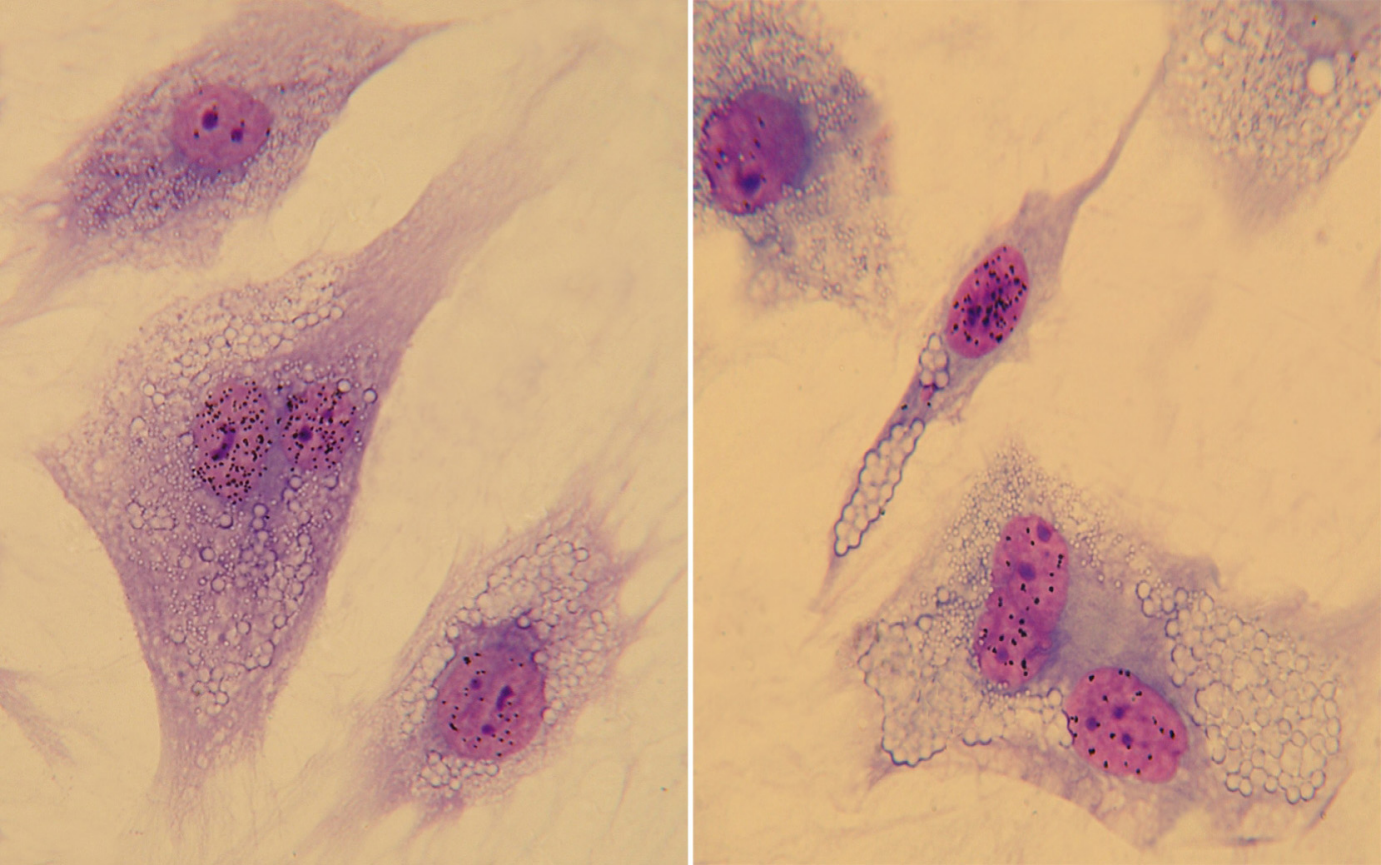
**Complementation test**. The classical complementation assay for nucleotide excision repair (NER) defects is based on the analysis of unscheduled DNA synthesis (UDS) in heterodikaryons obtained following fusion of primary dermal fibroblasts of the patient under study with cells representative of each of the various XP groups. To easily identify the fusion products, the two cell strains used as partners in the fusion are labelled with beads of different size. The two cell strains are classified in the same complementation group if the heterodikaryons, identified as binuclear cells containing beads of different sizes, fail to recover normal UDS levels and remain at the low levels seen in the mononuclear cells (right panel). Conversely, the restoration of normal UDS levels in the heterodikaryons (left panel) indicates that the cell strains used as partners in the fusion have genetically different defects (courtesy of Tiziana Nardo, IGM CNR Pavia).

Further tests can identify the gene defective in patients (complementation analysis) (Figure 2) (eg see [18]) and the causative mutation. This can give further insight into the clinical features but at present is not offered routinely as part of the diagnosis.

### Differential diagnosis

In severe cases, the diagnosis should be unequivocal. In milder cases, however, diagnosis can be much less clear-cut, with pigmentation changes not appearing until adolescence or even later. Solar urticaria can be excluded by the fact that the rash resolves within an hour of going indoors. Erythropoietic protoporphyria is easily screened through the finding of normal porphyrins and not every exposed skin site is affected in polymorphic light eruption. The sun-sensitivity of Cockayne Syndrome is manifest as an erythemal rash without the associated pigmentation changes that are typical of XP, and is associated with cachectic dwarfism and severe intellectual deficiency. Likewise the sun-sensitive rash of Rothmund-Thompson Syndrome is not associated with the pigmentation changes associated with XP. The pigmented lesions of Carney complex and Leopard syndrome are not related to sun exposure and in Peutz-Jeghers syndrome the lentigines are perioral and acral. A family history should also exclude these autosomal dominant lentiginoses.

### Genetic counselling

As with all genetic disorders, genetic counselling and psychological support is appropriate for the families, to discuss aetiology, probability of occurrence in future pregnancies, increased likelihood of occurrence in communities in which consanguineous marriages are common, feelings of isolation and concern about career prospects.

### Antenatal diagnosis

The DNA repair tests described above can be carried out on chorionic villus-derived cells or on amniocytes in affected families, as can molecular analysis if the mutation in the proband has been identified.

### Management and treatment

Although no cure is available for XP, the skin problems can be dramatically ameliorated by appropriate protection. However, some effects of sun exposure before diagnosis may appear years later despite adequate sun protection after diagnosis. As the skin changes are all caused by UV light, complete protection from exposure to UV can prevent further skin changes completely. Protective measures include the following:

(1) All windows in the home, car and school should be covered with UV-resistant film, which is commercially available. Hospital theatre lights, halogen lights, metal halide lamps and some fluorescent lights need to be avoided or covered.

(2) When outside during daylight hours, exposed skin should be covered with sunscreen and lip balm. Long trousers, long sleeves and gloves should be worn together with a UV-resistant face mask. If the patient finds this unacceptable, a broad-rimmed hat or "hoodie" and wrap-around sunglasses should be worn.

(3) Regular visits should be paid to the dermatologist, so that any pre-cancerous lesions can be removed as early as possible.

(4) Frequent eye examinations by an ophthalmologist are recommended.

(5) Rigorous sun-protection is likely to result in vitamin D deficiency, so vitamin D supplements should be prescribed.

(6) Patients should avoid cigarette smoke and other environmental carcinogens.

(7) Psychosocial issues need to be addressed: social isolation from peers at school and at home is common; career prospects are thwarted and the meticulous photoprotection measures can lead to denial.

Routine audiometry, measurement of head circumference, assessment of gait and deep tendon reflex testing can usually serve as a screen for the presence of XP-associated neurologic abnormalities. Unfortunately, however, the cause of the neurological problems is not fully understood, and there is no known way of preventing them at the time of writing. Management of patients with neurological involvement can include use of hearing aids, physical therapy, occupational therapy and speech therapy.

There are XP support groups in UK [19], USA [20], France [21] and Germany [22]. They offer a wealth of advice and help.

In the USA and UK, XP multidisciplinary clinics have been established - XP patients are seen by dermatology, ophthalmology, neurology, psychology, genetic and nursing specialists. These clinics are available to anyone diagnosed with XP or with a possible diagnosis of XP.

### Prognosis

For patients without neurological abnormalities, who are diagnosed early and carry out stringent protection measures as indicated above, the prognosis is good. They can expect a relatively normal lifespan, but will need to maintain their protection throughout their lives. The neurological abnormalities are progressive, leading to progressive disabilities, which will vary in severity between patients and are likely to result in a shortened lifespan.

## Conclusions

Although there is no cure for XP, increased awareness and crucially early diagnosis, followed by rigorous protection from daylight and careful patient management, can dramatically improve the quality of life and life expectancy of affected individuals. A major challenge for the future is to increase understanding of the aetiology of the neurological problems associated with the disorder in some individuals. This should enable the development of treatments to alleviate the neurological degeneration.

## Competing interests

The authors declare that they have no competing interests.

## Authors' contributions

All authors contributed to writing the manuscript. DM provided clinical expertise and ARL and MS provided cellular and molecular expertise. The figures are from the MS laboratory. All authors read and approved the final manuscript.

## References

[B1] Robbins JH Kraemer KH Lutzner MA Festoff BW Coon HG Xeroderma pigmentosum: an inherited disease with sun-sensitivity, multiple cutaneous neoplasms, and abnormal DNA repair Annals Internal Med 1974 80 221 248 10.7326/0003-4819-80-2-221 4811796

[B2] Hirai Y Kodama Y Moriwaki S Noda A Cullings HM Macphee DG Kodama K Mabuchi K Kraemer KH Land CE Nakamura N Heterozygous individuals bearing a founder mutation in the XPA DNA repair gene comprise nearly 1% of the Japanese population Mutat Res 2006 601 171 178 16905156 10.1016/j.mrfmmm.2006.06.010

[B3] Kleijer WJ Laugel V Berneburg M Nardo T Fawcett H Gratchev A Jaspers NG Sarasin A Stefanini M Lehmann AR Incidence of DNA repair deficiency disorders in western Europe: Xeroderma pigmentosum, Cockayne syndrome and trichothiodystrophy DNA Repair (Amst) 2008 7 744 750 10.1016/j.dnarep.2008.01.014 18329345

[B4] Kraemer KH Lee MM Scotto J Xeroderma Pigmentosum. Cutaneous, ocular and neurologic abnormalities in 830 published cases Archives of Dermatology 1987 123 241 250 10.1001/archderm.123.2.241 3545087

[B5] Stefanini M Kraemer KHK Ruggieri M, Pascual-Castroviejo I, Di Rocco C Xeroderma pigmentosum Neurocutaneous Diseases 2008 Chapter 51 771 792

[B6] Bradford PT Goldstein AM Tamura D Khan SG Ueda T Boyle J Oh KS Imoto K Inui H Moriwaki SI Emmert S Pike KM Raziuddin A Plona TM Digiovanna JJ Tucker MA Kraemer KH Cancer and neurologic degeneration in xeroderma pigmentosum: long term follow-up characterises the role of DNA repair J Med Genet 2011 48 168 176 10.1136/jmg.2010.083022 21097776 PMC3235003

[B7] Ramkumar HL Brooks BP Cao X Tamura D Digiovanna JJ Kraemer KH Chan CC Ophthalmic manifestations and histopathology of xeroderma pigmentosum: two clinicopathological cases and a review of the literature Surv Ophthalmol 2011 56 348 361 10.1016/j.survophthal.2011.03.001 21684361 PMC3137889

[B8] Andrews AD Barrett SF Robbins JH Xeroderma pigmentosum neurological abnormalities correlate with colony-forming ability after ultraviolet radiation Proceedings of the National Academy of Sciences of the United States of America 1978 75 1984 1988 10.1073/pnas.75.4.1984 273925 PMC392467

[B9] Lehmann AR Kirk-Bell S Arlett CF Paterson MC Lohman PHM de Weerd-Kastelein EA Bootsma D Xeroderma pigmentosum cells with normal levels of excision repair have a defect in DNA synthesis after UV-irradiation Proceedings of the National Academy of Sciences of the United States of America 1975 72 219 223 10.1073/pnas.72.1.219 1054497 PMC432274

[B10] Masutani C Kusumoto R Yamada A Dohmae N Yokoi M Yuasa M Araki M Iwai S Takio K Hanaoka F The XPV (xeroderma pigmentosum variant) gene encodes human DNA polymerase eta Nature 1999 399 700 704 10.1038/21447 10385124

[B11] Daya-Grosjean L Sarasin A The role of UV induced lesions in skin carcinogenesis: an overview of oncogene and tumor suppressor gene modifications in xeroderma pigmentosum skin tumors Mutat Res 2005 571 43 56 15748637 10.1016/j.mrfmmm.2004.11.013

[B12] Brooks PJ The 8, 5'-cyclopurine-2'-deoxynucleosides: candidate neurodegenerative DNA lesions in xeroderma pigmentosum, and unique probes of transcription and nucleotide excision repair DNA Repair (Amst) 2008 7 1168 1179 10.1016/j.dnarep.2008.03.016 PMC2797313 18495558

[B13] Stefanini M Keijzer W Dalpra L Elli R Porro MN Nicoletti B Nuzzo F Differences in the levels of UV repair and in clinical symptoms in two sibs affected by xeroderma pigmentosum Hum Genet 1980 54 177 182 10.1007/BF00278968 7390491

[B14] Lehmann AR Stevens S A rapid procedure for measurement of DNA repair in human fibroblasts and for complementation analysis of xeroderma pigmentosum cells Mutation Research 1980 69 177 190 6987495 10.1016/0027-5107(80)90187-6

[B15] Limsirichaikul S Niimi A Fawcett H Lehmann A Yamashita S Ogi T A rapid non-radioactive technique for measurement of repair synthesis in primary human fibroblasts by incorporation of ethynyl deoxyuridine (EdU) Nucleic Acids Res 2009 37 e31 19179371 10.1093/nar/gkp023 PMC2651789

[B16] Arlett CF Harcourt SA Broughton BC The influence of caffeine on cell survival in excision-proficient and excision-deficient xeroderma pigmentosum and normal human cell strains following ultraviolet light irradiation Mutation Research 1975 33 341 346 1214825 10.1016/0027-5107(75)90209-2

[B17] Broughton BC Cordonnier A Kleijer WJ Jaspers NG Fawcett H Raams A Garritsen VH Stary A Avril MF Boudsocq F Masutani C Hanaoka F Fuchs RP Sarasin A Lehmann AR Molecular analysis of mutations in DNA polymerase eta in xeroderma pigmentosum-variant patients Proc Natl Acad Sci USA 2002 99 815 820 10.1073/pnas.022473899 11773631 PMC117388

[B18] Chavanne F Broughton BC Pietra D Nardo T Browitt A Lehmann AR Stefanini M Mutations in the *XPC *gene in families with xeroderma pigmentosum and consequences at the cell, protein and transcript levels Cancer Research 2000 60 1974 1982 10766188

[B19] Xeroderma pigmentosum support group http://joomla.xpsupportgroup.org.uk

[B20] XP family support group http://www.xpfamilysupport.org and xeroderma pigmentosum society http://www.xps.org/

[B21] Enfants de la lune http://asso.orpha.net/AXP

[B22] XP Freu(n)de http://www.xerodermapigmentosum.de

[B23] Human DNA repair genes http://sciencepark.mdanderson.org/labs/wood/DNA_Repair_Genes.html

